# Case report of an unusual allergic reaction to a routine skin prick test performed in an outpatient clinic: Diagnosis, management, and knowledge gaps

**DOI:** 10.1097/MD.0000000000038628

**Published:** 2024-07-05

**Authors:** Karla Robles-Velasco, Denisse Cevallos-Levicek, Giselle Mosnaim, Jie Shen Fok, Ivan Cherrez-Ojeda

**Affiliations:** a Universidad Espíritu Santo, Samborondón, Ecuador; b Respiralab, Respiralab Research Group, Guayaquil, Ecuador; c Division of Allergy and Immunology, Department of Medicine, NorthShore University Health System, Evanston, Illinois, USA; d Department of Respiratory Medicine and General Medicine, Box Hill Hospital, Melbourne, Australia; e Monash Lung, Sleep and Allergy/Immunology, Monash Medical Centre, Melbourne, Australia; f Eastern Health Clinical School, Monash University, Melbourne, Australia.

**Keywords:** allergic reaction, allergy, dust mites, skin prick test, type 1 hypersensitivity reaction

## Abstract

**Background:**

The skin prick test (SPT) is a standard procedure in allergy/immunology clinics, crucial for evaluating conditions like allergic rhinitis and food allergies. As a cornerstone in investigating immunoglobulin E-mediated allergy, it plays a vital role in diagnosing allergies, including those triggered by common dust mites like *Dermatophagoides pteronyssinus, Dermatophagoides farinae, Euroglyphus maynei*, and *Blomia tropicalis*. Despite its widespread use, adverse reactions to SPT are uncommon (15 per 100,000 patients), though the procedure is not entirely risk-free. This article presents a clinical case involving a 17-year-old female who experienced a moderately delayed allergic reaction 120 minutes post-SPT, managed effectively with subsequent symptom resolution.

**Methods:**

The patient, with a history of persistent rhinorrhea, itchy nose, eyes, and postnasal drip, sought consultation due to worsening symptoms. Diagnostic measures, including patient-reported outcomes and SPT with a standard aeroallergen panel, revealed sensitization to various allergens.

**Results:**

Post-test, the patient reported ocular pruritus, left eyelid swelling, and moderate rhinorrhea, persisting for about 24 hours. On the subsequent medical visit, the patient received rupatadine and deflazacort, leading to symptom resolution within 3 hours.

**Conclusion:**

This article delves into a systemic allergic reaction post-SPT, emphasizing the 2 main stages of type I hypersensitivity reactions. While the acute phase involves mast cell-driven mediators within 15 minutes, the delayed phase (4–8 hours) includes de novo cytokine release. Vigilance regarding symptom onset and differentiation between mild and severe reactions is crucial. Notably, the absence of specific waiting time guidelines post-SPT underscores the need for reporting to enhance understanding and subsequent management. Performing these procedures in specialized centers with qualified professionals is essential for effectively managing potential anaphylactic reactions. Addressing these knowledge gaps will contribute to enhanced patient safety during diagnostic procedures.

## 1. Introduction

Skin prick test (SPT) is a routine procedure in allergy/immunology clinics. It is widely utilized in the assessment of allergic rhinitis and food allergy.^[1]^ It is the cornerstone in investigating immunoglobulin E (IgE)-mediated allergy.^[1]^ The principle of SPT involves the activation of IgE antibodies in cutaneous mast cells when they are exposed to specific allergens contained in allergen extracts which come in the form of liquid droplets and are pricked using a sterile lancet. Histamine is then released by mast cells, resulting in a wheal response usually measured in 15 minutes.^[2]^

SPT has good sensitivity and specificity to aeroallergens, approximately 70 to 95% and 80 to 97%, respectively.^[3]^ Dust mites are a common allergen globally, causing allergic rhinitis, allergic conjunctivitis, allergic asthma, and atopic dermatitis,^[4]^ with *Dermatophagoides pteronyssinus, Dermatophagoides farinae, Euroglyphus maynei*, and *Blomia tropicalis* being the most prevalent and studied species.^[5]^

The probability of an adverse reaction after a SPT is unusual (15 per 100,000 patients).^[6]^ SPT is not risk-free, though adverse events are rare. Hence, our objective is to provide a clinical case involving a female patient who experienced a moderately delayed allergic reaction after 120 minutes of SPT, which subsequently resolved with effective symptom management.

## 2. Case report

**Figure 1.**
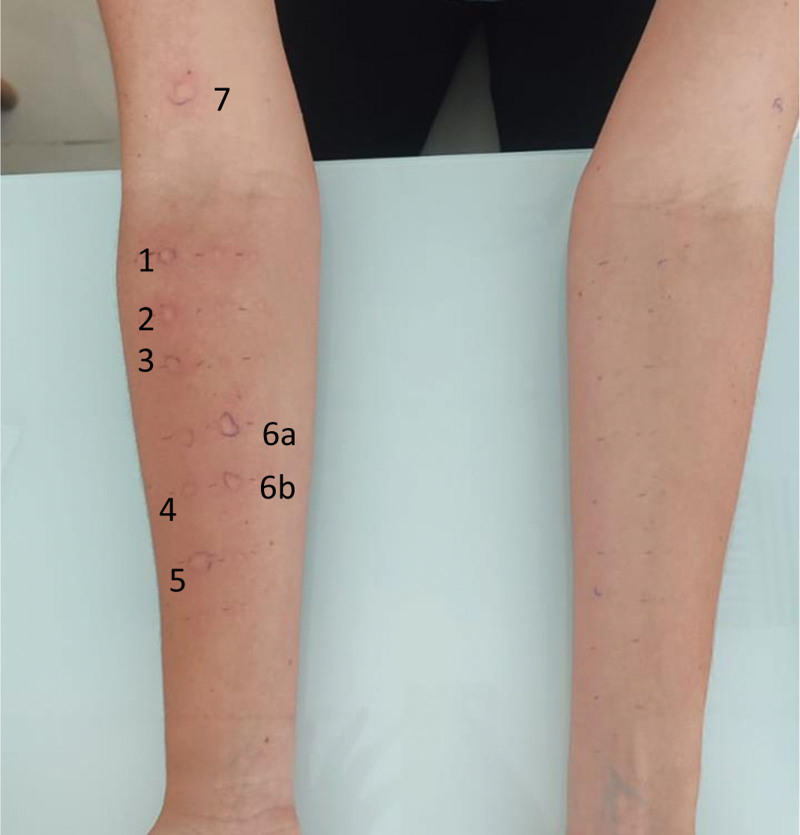
Skin prick test using commercial extracts, positive to (1) *Dermatophagoides Farinae*, (2) *Dermatophagoides Pteronyssinus*, (3) *Blomia Tropicalis*, (4) *Acarus siro*, (5) *Euroglyphus maynei*, (6a–b) Dog dander, (7) Histamine control.

A 17-year-old Hispanic female university student presented with long-standing rhinorrhea, itchy nose, itchy eyes, and postnasal drip. Her medical history included deviated septum and rhinitis since childhood, which was managed with oral antihistamines. She sought consultation with an allergy and immunology specialist because of the worsening of her clinical condition. Two patient-reported outcome measures were performed, including the Rhinitis Control test with a score of 16 (uncontrolled) and the Rhinoconjunctivitis Quality of Life Questionnaire with a score of 88 (moderate impact on quality of life). As part of the standard allergy evaluation, SPT was carried out with a standard aeroallergen panel using commercial extracts (Immunotek, Spain). SPT demonstrated evidence of sensitization to *Dermatophagoides Farinae, Dermatophagoides Pteronyssinus, Blomia Tropicalis, Acarus siro, Euroglyphus maynei*, and dog dander (Fig. 1). She was kept under observation for 30 minutes and discharged with a prescription for oral antihistamines. A subcutaneous immunotherapy was planned for the next visit.

**Figure 2.**
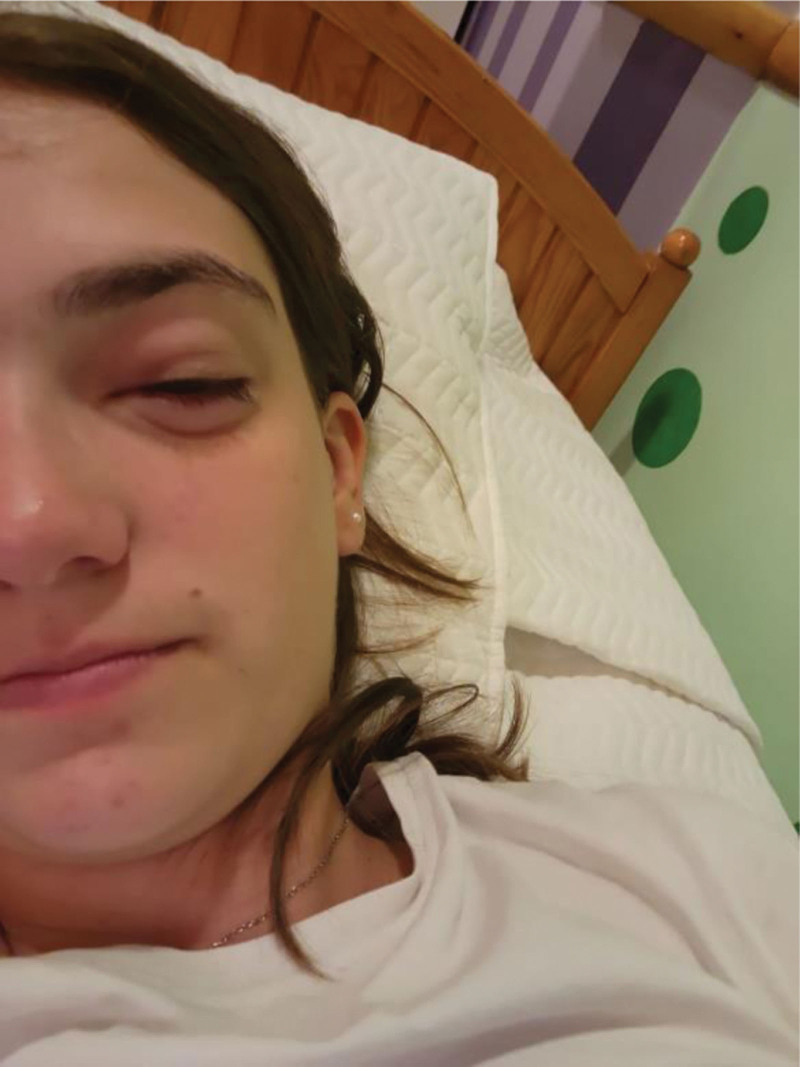
Selfie of the patient with left eyelid edema taken 3 hours after SPT. SPT = skin prick test.

About 2 hours after SPT, she reported ocular pruritus. Left eyelid swelling was subsequently observed 30 minutes later (Fig. 2), along with symptoms of moderate rhinorrhea and nasal congestion. Eyelid edema was still present until the following day.

On a medical visit the following day, the patient was instructed to take 10 mg of rupatadine every 12 hours for 5 days and deflazacort 6 mg once a day for 3 days, followed by 3 mg once a day for 4 days. Within 3 hours, she achieved symptom resolution.

## 3. Discussion

This article describes a systemic allergic reaction to SPT that arises through a series of events associated with type I hypersensitivity reactions, which consists of 2 main stages. The first acute phase, which occurs within 15 minutes, involves mast cell-driven mediators that are immediately released into the blood. The delayed phase, occurring within 4 to 8 hours, involves the release of cytokines such as interleukin (IL)-1, IL-4, IL-5, IL-13, tumor necrosis factor, and granulocyte monocyte colony-stimulating factor produced de novo by mast cells.^[6]^

Physicians should be aware of the time of symptom onset, discriminate between mild and potentially life-threatening reactions, and promptly manage untoward events. However, to the best of our knowledge, there is no current SPT guideline that rules the specific waiting time after performing an SPT. Therefore, it is essential to report these reactions to enhance our understanding of the potential occurrence of these serious events following an ambulatory SPT and its subsequent management and follow-up.

Dust mite allergy is a global health problem recognized by the World Health Organization, which affects millions of people around the world and is a cause of absenteeism and reduced productivity in the workplace.^[5]^ The SPT is considered the most appropriate method for diagnosing allergy to aeroallergens^[7]^ and systemic allergic reaction rates to this test have been reported to be low, such as 23 reactions per 100,000 aeroallergen skin tests,^[8]^ these reactions are generally mild, immediate, and respond to treatment with standard measures.^[9]^ A recent study estimated the risk of systemic reactions to be between 0.008% and 0.12%.^[10,11]^

Allergic reactions after SPT are due to a massive release of inflammatory mediators, such as histamine, which is found in abundance in mast cells and basophils.^[12]^ Histamine causes increased permeability and dilatation of blood vessels, and leakage of intravascular fluid into the surrounding tissues. This resulted in skin flushing and angioedema.^[7]^ There are also reports of the relationship between house dust mites and MRGPRX2, a molecule involved in IgE-independent neuroimmunomodulation of itch and allergy response^[13]^ and Kumar et al^[14]^ have reported potential therapeutic inhibitory molecules to tackle the activation of MRGPRX2. In the case of an adverse event to a routine SPT, where there is typically a skin reaction mediated by IgE (with results reported as positive or negative),^[7]^ it is possible that MRGPRX2 has an underlying role in these reactions. However, the patient in question did not have a past medical history of atopic dermatitis, asthma, or a Th2-type spectrum. Her generalized reaction included a moderate clinical picture of rhinorrhea, nasal congestion, and eyelid edema, without skin involvement in the area intervened with the SPT. This potential involvement of MRGPRX2 in non-IgE-mediated reactions underscores the importance of identifying this target.^[15, 16]^ Doing so could inform future guidelines for premedication targeting these specific molecules before a standard SPT, potentially preventing unexpected outcomes.

The subsequent symptoms are determined by the route and location of the allergen exposure. The presence of inhaled allergens has the potential to worsen symptoms of allergic rhinitis or asthma, leading to nasal congestion, rhinorrhea, sneezing, and bronchospasm.^[17,18]^ Urticaria can be induced by direct exposure to allergens.^[19]^ Moreover, the induction of systemic symptoms is commonly observed when individuals are exposed to allergens by oral or intravenous routes.^[20]^In the present scenario, the patient under consideration exhibited the occurrence of urticaria and angioedema after topical contact.

The first step in treating allergic reactions is to evaluate the severity. The World Allergy Organization (WAO) classifies reactions as grade 2 if ≥2 organ systems are involved.^[20]^ The European Academy of Allergy, Asthma and Immunology Ocular Allergy Interest Group on Diagnosis and Management of Ocular Allergy recommends the use of second-generation H1 antihistamines for patients with allergic conjunctivitis and nasal symptoms.^[21]^ In reactions involving allergic conjunctivitis, oral corticosteroids are recommended in extreme cases that cannot be controlled with standard treatments.^[3,5]^

Treatment plan for this patient was in line with current guidelines. Epinephrine was deemed not required, especially in the absence of cardiovascular or gastrointestinal compromise or involvement. Standard doses of second-generation H1-antihistamines are sufficient to manage ocular and nasal symptoms, including eye angioedema. In this case, injectable epinephrine was not used because the symptoms were not within the category of anaphylaxis according to the “Amended criteria for the diagnosis of anaphylaxis, proposed by the WAO Anaphylaxis Committee, 2019.” In this WAO report, anaphylaxis is very likely when one of the following 2 criteria are met. Criteria 1 includes acute onset of an illness (from minutes to several hours) with involvement of the skin, mucosal tissue, or both with at least one of the following symptoms: respiratory compromise; decreased blood pressure or associated symptoms of end-organ dysfunction; severe gastrointestinal symptoms. Criteria 2 includes acute onset of hypotension or bronchospasm or laryngeal involvement after exposure to a known allergen (minutes to several hours).^[22]^

The objective of our case presentation was to provide information about the prospective adverse effects linked to traditional ambulatory SPTs and to emphasize the possible gap in knowledge regarding the necessary monitoring time for patients immediately after an SPT as well as the potential characteristics and main risk factors of patients who may experience an adverse reaction after SPT. These shortcomings will be addressed by further investigation of this subject. It is crucial to point out the significance of conducting this diagnostic procedure at specialized centers staffed by qualified professionals, such as allergists and immunologists, who have the required expertise and resources to handle anaphylactic reactions, including immediate access to injectable epinephrine.

## Author contributions

**Conceptualization:** Denisse Cevallos-Levicek, Karla Robles-Velasco, Ivan Cherrez-Ojeda.

**Formal analysis:** Denisse Cevallos-Levicek, Karla Robles-Velasco.

**Investigation:** Denisse Cevallos-Levicek, Karla Robles-Velasco, Jie Shen Fok.

**Methodology:** Denisse Cevallos-Levicek, Karla Robles-Velasco, Giselle Mosnaim, Jie Shen Fok.

**Project administration:** Ivan Cherrez-Ojeda.

**Supervision:** Karla Robles-Velasco, Ivan Cherrez-Ojeda, Giselle Mosnaim, Jie Shen Fok.

**Validation:** Karla Robles-Velasco, Ivan Cherrez-Ojeda, Giselle Mosnaim, Jie Shen Fok.

**Visualization:** Denisse Cevallos-Levicek, Giselle Mosnaim, Jie Shen Fok.

**Writing – original draft:** Denisse Cevallos-Levicek, Karla Robles-Velasco, Ivan Cherrez-Ojeda, Giselle Mosnaim, Jie Shen Fok.

**Writing – review & editing:** Denisse Cevallos-Levicek, Karla Robles-Velasco, Ivan Cherrez-Ojeda, Giselle Mosnaim, Jie Shen Fok.
